# Tert-butylhydroquinone attenuates doxorubicin-induced dysregulation of testicular cytoprotective and steroidogenic genes, and improves spermatogenesis in rats

**DOI:** 10.1038/s41598-021-85026-7

**Published:** 2021-03-09

**Authors:** Godwin Adakole Ujah, Victor Udo Nna, Joseph Bagi Suleiman, Chinedum Eleazu, Chukwuemeka Nwokocha, Joy Assima Rebene, Michael Umana Imowo, Emmanuel Ochui Obi, Charlette Amachree, Evarest Chigozie Udechukwu, Mahaneem Mohamed

**Affiliations:** 1 Department of Physiology, College of Medical Sciences, University of Calabar, P.M.B. 1115, Calabar, Cross River State Nigeria; 2 Department of Physiology, School of Medical Sciences, Universiti Sains Malaysia, 16150 Kubang Kerian, Kelantan Malaysia; 3 Department of Science Laboratory Technology, Akanu Ibiam Federal Polytechnic, Unwana, Afikpo, Ebonyi State Nigeria; 4 Department of Chemistry, Biochemistry and Molecular Biology, Alex Ekwueme Federal University, Ndufu-Alike, Ikwo, Ebonyi State Nigeria; 5 Department of Basic Medical Sciences (Physiology Section), The University of the West Indies, Mona, Kingston 7, Jamaica; 6 Unit of Integrative Medicine, School of Medical Sciences, Universiti Sains Malaysia, 16150 Kubang Kerian, Kelantan Malaysia

**Keywords:** Endocrine reproductive disorders, Infertility

## Abstract

Doxorubicin (DOX) is a broad-spectrum chemotherapeutic drug used in the treatment of cancers. It acts by generating reactive oxygen species in target cells. The actions are, however, not limited to cancerous cells as it attacks healthy cells, killing them. This study investigated the benefits of the antioxidant, tert-butylhydroquinone (tBHQ), on testicular toxicity following DOX therapy. Twenty-four adult male albino rats were assigned randomly into four groups (n = 6), namely: normal control (NC), tBHQ, DOX and tBHQ + DOX groups. tBHQ (50 mg/kg body weight in 1% DMSO) was administered orally for 14 consecutive days, while a single DOX dose (7 mg/kg body weight) was administered intraperitoneally on Day 8. DOX decreased sperm count, motility and viability, and decreased the levels of steroidogenesis-related proteins, and reproductive hormones. Furthermore, DOX decreased the expression of antioxidant cytoprotective genes, and decreased the protein level of proliferating cell nuclear antigen in the testis. Conversely, DOX increased the expression of pro-inflammatory and pro-apoptotic genes in the testis. These negative effects were ameliorated following the intervention with tBHQ. Our results suggest that tBHQ protects the testis and preserves both steroidogenesis and spermatogenesis in DOX-treated rats through the suppression of oxidative stress, inflammation and apoptosis.

## Introduction

Doxorubicin (DOX) is a common chemotherapeutic drug that is used to treat a wide range of cancers such as gastric, lung, breast, thyroid, ovarian, testicular, leukaemia, neuroblastoma, malignant lymphoma, multiple myeloma and sarcoma^1^. It has been in use for decades^2^, and compared to other chemotherapeutic drugs, has a broader spectrum^3^, making it one of the most prescribed chemotherapeutic drugs^4^. The anticancer property of DOX results from its intercalation into DNA, disruption of DNA synthesis by inhibiting topoisomerase II^5^, and production of reactive oxygen species (ROS), leading to the damage of cell membranes, DNA and proteins, thus resulting in the regulation of cell proliferation and death^6,7^. However, the effects of DOX are not limited to cancerous cells, but also healthy proliferating cells like the male germ cells^8,9^.

The membranes of the male germ cells are enriched with polyunsaturated fatty acids which is, unfortunately, one of the targets of ROS^10^. Therefore, spermatozoa are susceptible to oxidative damage because of the high amount of lipids in their membranes, and this makes them lose their integrity and become less motile^10,11^. DOX has been shown to impair male fertility by causing germ cell oxidative stress and apoptosis^12–14^. It has also been demonstrated to impair spermatogenesis^13,15^ and steroidogenesis^16,17^. Exposure to DOX seems to affect testicular integrity at both prepubertal and post-pubertal stages of development. In vitro studies that employed prepubertal mouse testis demonstrated significant loss in germ cell number following exposure to DOX at concentrations that were equivalent to human therapeutic doses^18^. Further, studies have demonstrated early testicular developmental arrest and long-term germ cell DNA damage following prepubertal DOX exposure^19,20^. Studies using adult rat models of DOX exposure have reported decreased testosterone, follicle stimulating hormone (FSH) and luteinizing hormone (LH) levels, decreased sperm count, motility and viability, and increased abnormally formed spermatozoa^16,17,21^.

One of the mechanisms by which ROS effects are mitigated, depends on nuclear factor erythroid-2-related factor 2 (Nrf2)-mediated activation of cellular antioxidant response element (ARE)^22–24^. AREs are cis-acting proteins mediating transcriptional activation of cellular genes exposed to ROS. They are essential in cellular defence against oxidative insults. They co-ordinate the upregulation of protective genes, that help mitigate the debilitating effects of ROS.

Tert-butylhydroquinone (tBHQ), a metabolite of butylated hydroxyanisole^25^, is known to activate Nrf2^26^. It enjoys wide usage as a synthetic food preservative, preventing oxidative deterioration of fats and oils^27^. It has also been shown to have antioxidant, anti-inflammatory and anti-apoptotic properties^28–30^. Studies have shown that tBHQ offers protection against ROS-mediated toxicity in hepatocytes^31^, astrocytes^32^, nephrons^33^, intestinal mucosa^30^ and lungs^34^. tBHQ has also been demonstrated to ameliorate doxorubicin-induced cardiotoxicity^35^. Given these properties of tBHQ and the knowledge that DOX impairs male fertility by ROS generation, this study evaluated the protective effects of tBHQ on DOX-induced testicular injury.

## Results

### Body weight gain, testicular and epididymal absolute and relative weights

DOX decreased (*p* < 0.05) both final body weight and body weight gain when compared to the normal control (NC) group. In combination with tBHQ, however, there was an increase (*p* < 0.05) in both parameters when compared to DOX group (Table 1). Furthermore, DOX decreased (*p* < 0.05) absolute and relative weights of both epididymis and testes when compared to the NC group. In combination with tBHQ, both parameters increased (*p* < 0.05) when compared with DOX group (Table 1).

**Table 1:** Body weight, testicular and epididymal absolute and relative weights.

Parameter	NC	tBHQ	DOX	tBHQ + DOX
Initial body weight (g)	228.70 ± 5.50	227.70 ± 7.23	229.50 ± 5.75	227.00 ± 6.51
Final body weight (g)	250.20 ± 4.71	252.20 ± 8.61	233.30 ± 5.89^a^	246.50 ± 7.29^c^
Body weight gain (%)	8.60 ± 1.26	9.71 ± 0.52	1.64 ± 0.56^a^	7.90 ± 1.37^c^
Absolute testes weight (g)	3.59 ± 0.20	3.70 ± 0.20	2.35 ± 0.13^a^	3.29 ± 0.22^c^
Absolute epididymal weight (g)	1.23 ± 0.13	1.25 ± 0.09	0.77 ± 0.10^a^	1.16 ± 0.12^c^
Relative testes weight (%)	1.43 ± 0.09	1.47 ± 0.10	1.01 ± 0.07^a^	1.34 ± 0.11^c^
Relative epididymal weight (%)	0.50 ± 0.06	0.50 ± 0.04	0.33 ± 0.05^a^	0.47 ± 0.05^c^

Values are mean ± SD, n = 6/group. NC: normal control, tBHQ: tert-butylhydroquinone, DOX: doxorubicin.

^a^*p* < 0.05 versus NC group, ^c^*p* < 0.05 versus DOX group (One-way ANOVA with Tukey’s post-hoc test).

### Histology of the testis

**Figure 1:**
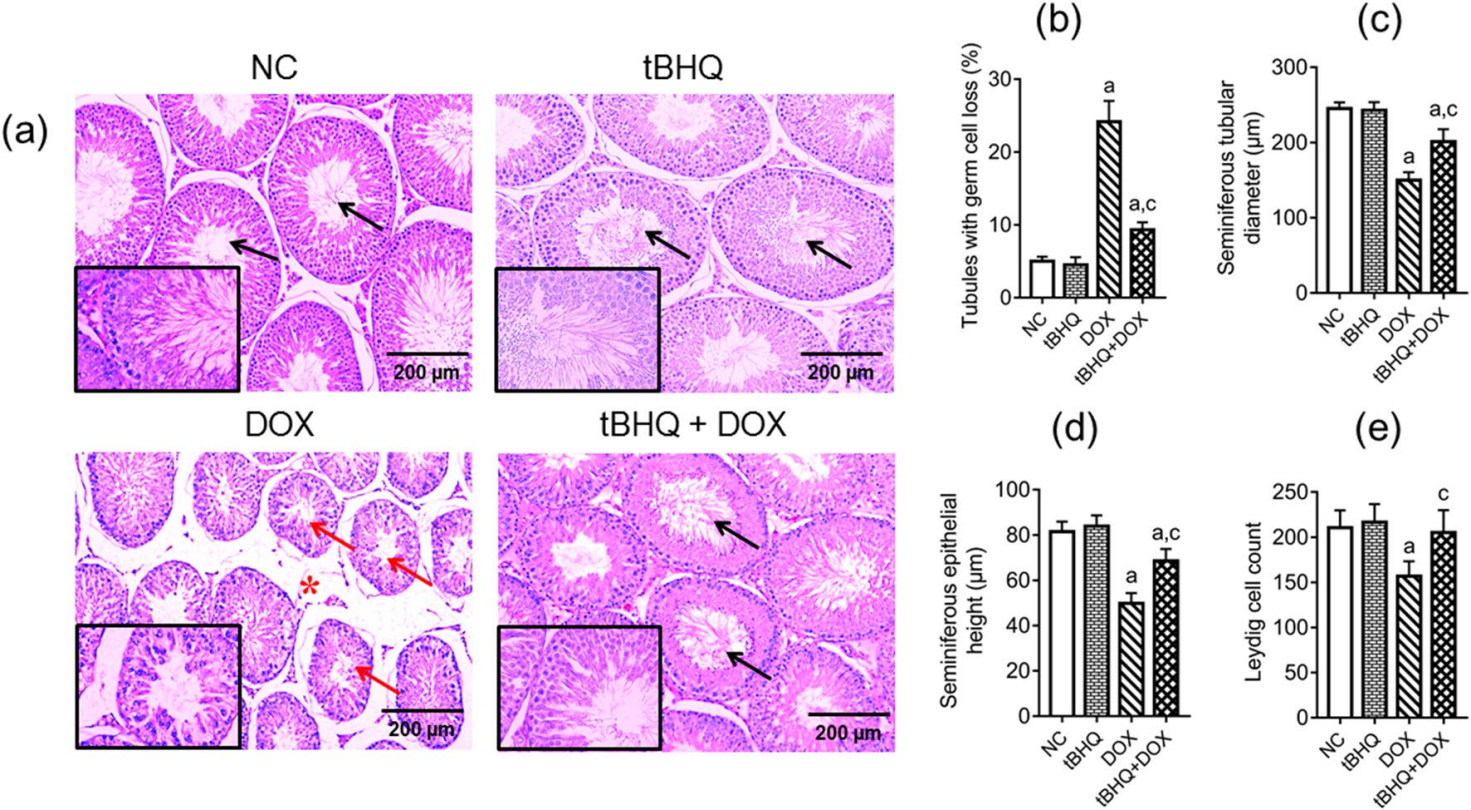
Effect of tBHQ on the testicular structure of DOX-treated rats. (**a**) Representative H&E staining light microscope images of testicular tissue (magnification: × 100, scale bar: 200 μm, and × 400, scale bar: 50 µm for representative images with black borders). DOX decreased seminiferous tubular diameter giving rise to large intertubular spaces (red asterisks). Numerous seminiferous tubules in DOX group had germ cell loss (red arrows) compared to the NC and tBHQ-treated groups showing several tubules with complete spermatogenesis and sperm cells in the lumen (black arrows). NC: normal control, tBHQ: tert-butylhydroquinone, DOX: doxorubicin, tBHQ + DOX: tert-butylhydroquinone + doxorubicin. Numerical data show seminiferous tubules with germ cell loss (**b**), seminiferous tubular diameter (**c**), seminiferous epithelial height (**d**), and Leydig cell count (**e**), respectively. Values are mean ± SD, n = 6/group. ^a^*p* < 0.05 versus NC; ^c^*p* < 0.05 versus DOX (One-way ANOVA with Tukey’s post-hoc test).

Following DOX administration, many seminiferous tubules decreased in size, leaving large intertubular spaces between adjourning seminiferous tubules (Fig. 1a). Numerical data showed that there was an increase (*p* < 0.05) in the percentage of seminiferous tubules with germ cell loss and decrease (*p* < 0.05) in tubular diameter in DOX group when compared to the NC group (Fig. 1b,c). Also, seminiferous tubular epithelial height decreased (*p* < 0.05) in DOX group relative to the NC group (Fig. 1d). This is reflected in Fig. 1a where there is significant spatial separation between the seminiferous tubules in DOX group as compared to the NC group. Leydig cell count reduced (*p* < 0.05) in DOX group when compared to the NC group (Fig. 1e). When tBHQ + DOX were co-administered, the percentage of tubules with germ cell loss reduced (*p* < 0.05), tubular diameter, epithelial height and Leydig cell count increased (*p* < 0.05) when compared with DOX group (Fig. 1b–e).

### Hormonal levels and steroidogenesis regulatory genes and protein levels

**Figure 2:**
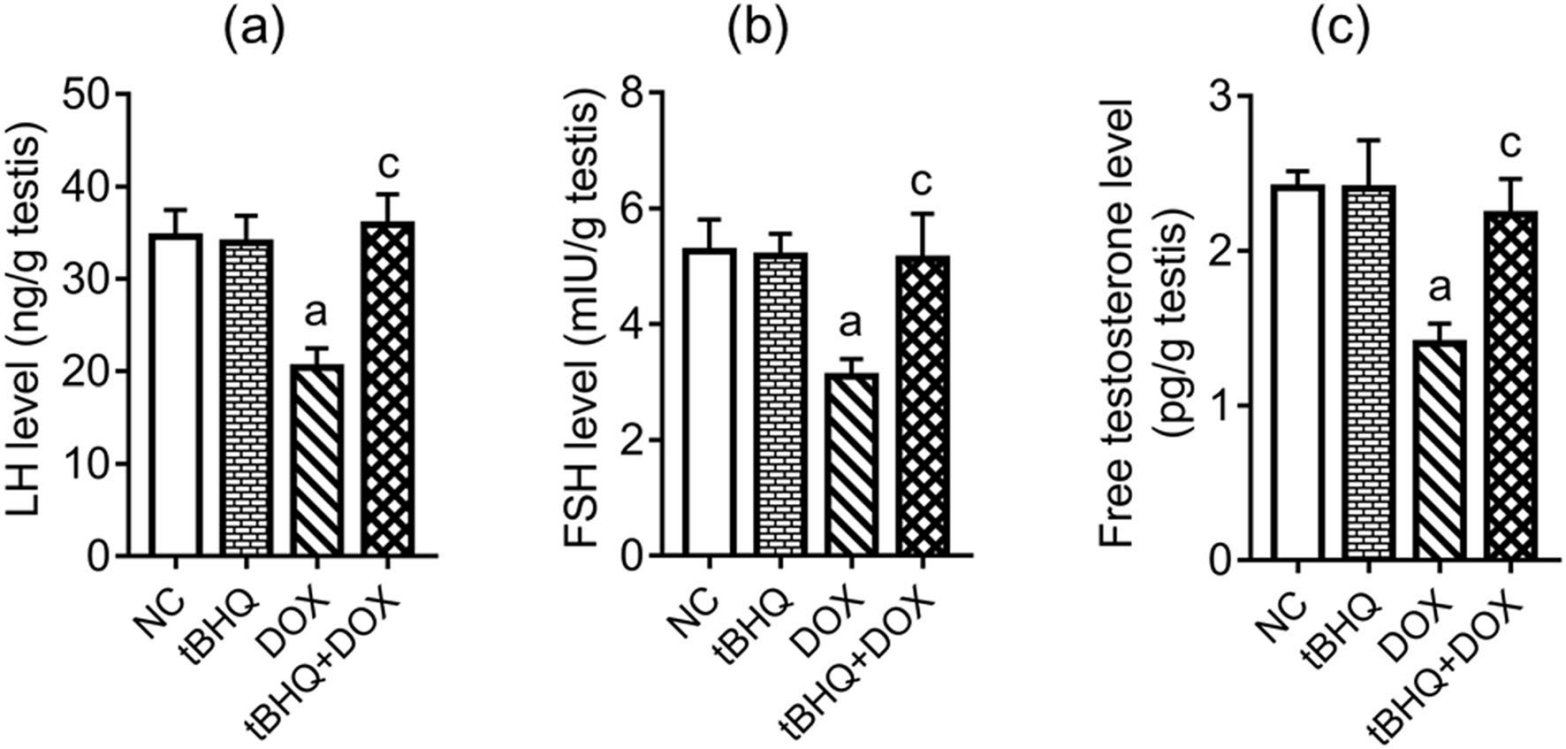
Effect of tBHQ on testicular levels of LH (**a**), FSH (**b**) and free testosterone (**c**) in DOX-treated rats. NC: normal control, tBHQ: tert-butylhydroquinone, DOX: doxorubicin, tBHQ + DOX: tert-butylhydroquinone + doxorubicin, FSH: follicle stimulating hormone, LH: luteinizing hormone. Values are mean ± SD, n = 6/group. ^a^*p* < 0.05 versus NC; ^c^*p* < 0.05 versus DOX (One-way ANOVA with Tukey’s post-hoc test).

**Figure 3:**
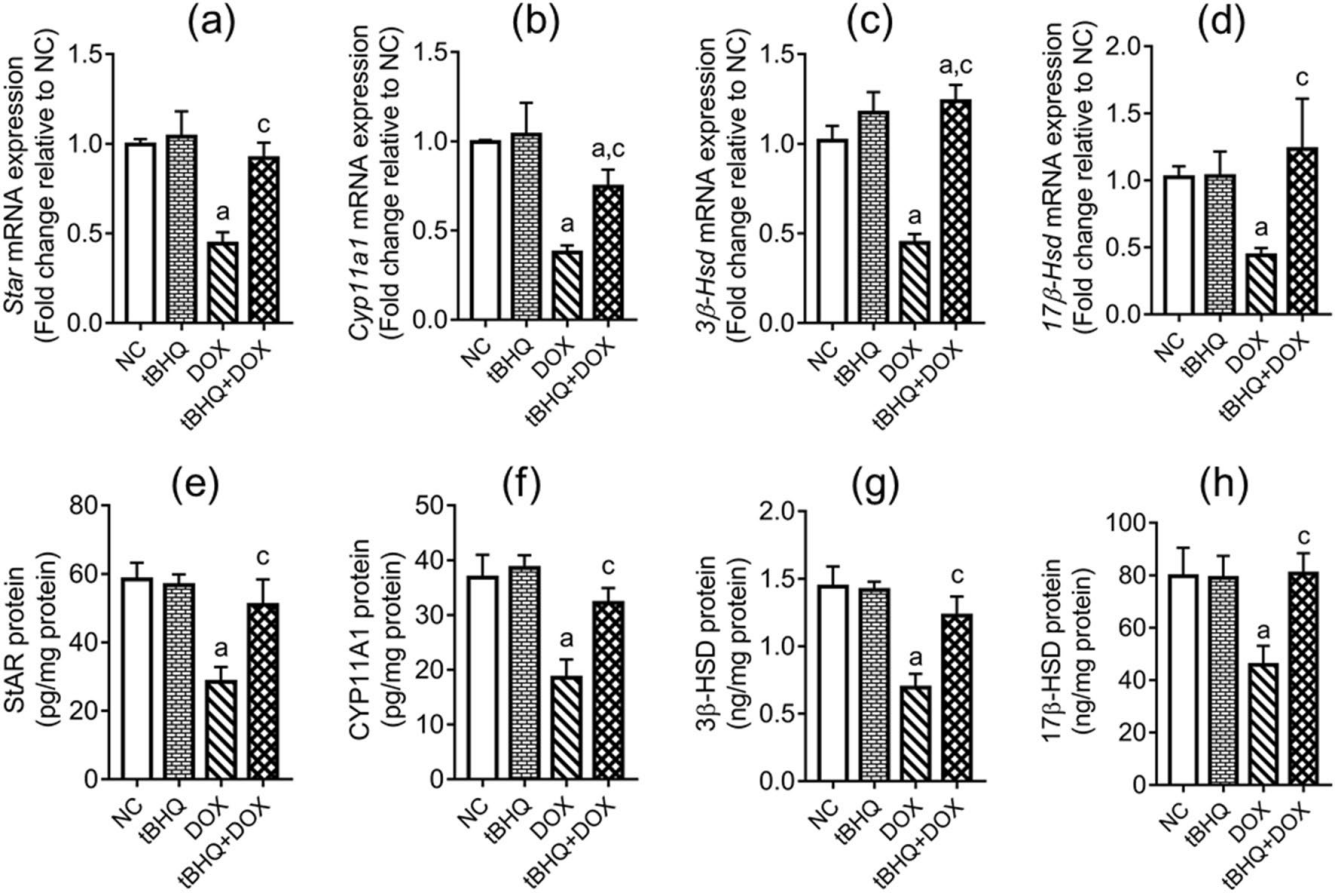
Effect of tBHQ on testicular mRNA and protein levels of StAR (**a**,**e**), CYP11A1 (**b**,**f**), 3β-HSD (**c**,**g**) and 17β-HSD (**d**,**h**) in DOX-treated rats. NC: normal control, tBHQ: tert-butylhydroquinone, DOX: Doxorubicin, tBHQ + DOX: tert-butylhydroquinone + doxorubicin, StAR: steroidogenic acute regulatory protein, CYP11A1: cholesterol side chain cleavage enzyme, HSD: hydroxysteroid dehydrogenase. Values are mean ± SD, n = 6/group. ^a^*p* < 0.05 versus NC; ^c^*p* < 0.05 versus DOX (One-way ANOVA with Tukey’s post-hoc test).

The levels of LH, FSH and testosterone in the testes decreased (*p* < 0.05) in DOX group compared to the NC group. However, pre-treatment with tBHQ prevented DOX effects on the levels of these hormones (Fig. 2a–c). DOX downregulated the expression of *Star*, *Cyp11a1*, *3β*-*Hsd* and *17β*-*Hsd* genes (Fig. 3a–d) in the testis and decreased (*p* < 0.05) their corresponding protein levels in the testis (Fig. 3e–h). However, tBHQ prevented (*p* < 0.05) DOX effects on the steroidogenic genes and their corresponding protein levels (Fig. 3a–h).

### Sperm analysis

DOX decreased (*p* < 0.05) sperm count, motility, sperm viability and Johnsen score, and increased (*p* < 0.05) the percentage of sperm with abnormal morphology compared to the NC group (Table 2). However, tBHQ ameliorated DOX effects on sperm count, viability and motility, and prevented DOX effects on sperm morphology and Johnsen score (Table 2).

**Table 2:** Sperm parameters in the different experimental groups.

Parameter	NC	tBHQ	DOX	tBHQ + DOX
Sperm count (× 10^6^/mL)	48.10 ± 5.35	48.25 ± 3.49	22.62 ± 3.77^a^	35.33 ± 4.08^a,c^
Sperm viability (%)	84.83 ± 5.67	85.17 ± 5.46	48.00 ± 5.97^a^	72.00 ± 7.40^a,c^
Sperm motility (%)	82.17 ± 5.08	82.67 ± 4.80	39.67 ± 3.14^a^	61.00 ± 4.47^a,c^
Abnormal morphology (%)	13.17 ± 3.06	12.83 ± 3.19	49.83 ± 7.17^a^	19.00 ± 2.37^c^
Mean Johnsen Score	9.50 ± 0.55	9.83 ± 0.41	4.83 ± 0.75^a^	8.50 ± 1.05^c^

Values are mean ± SD, n = 6/group. NC: normal control, tBHQ: tert-butylhydroquinone, DOX: doxorubicin.

^a^*p* < 0.05 versus NC group, ^c^*p* < 0.05 versus DOX group (One-way ANOVA with Tukey’s post-hoc test).

### Testicular expression of antioxidant genes

**Figure 4:**
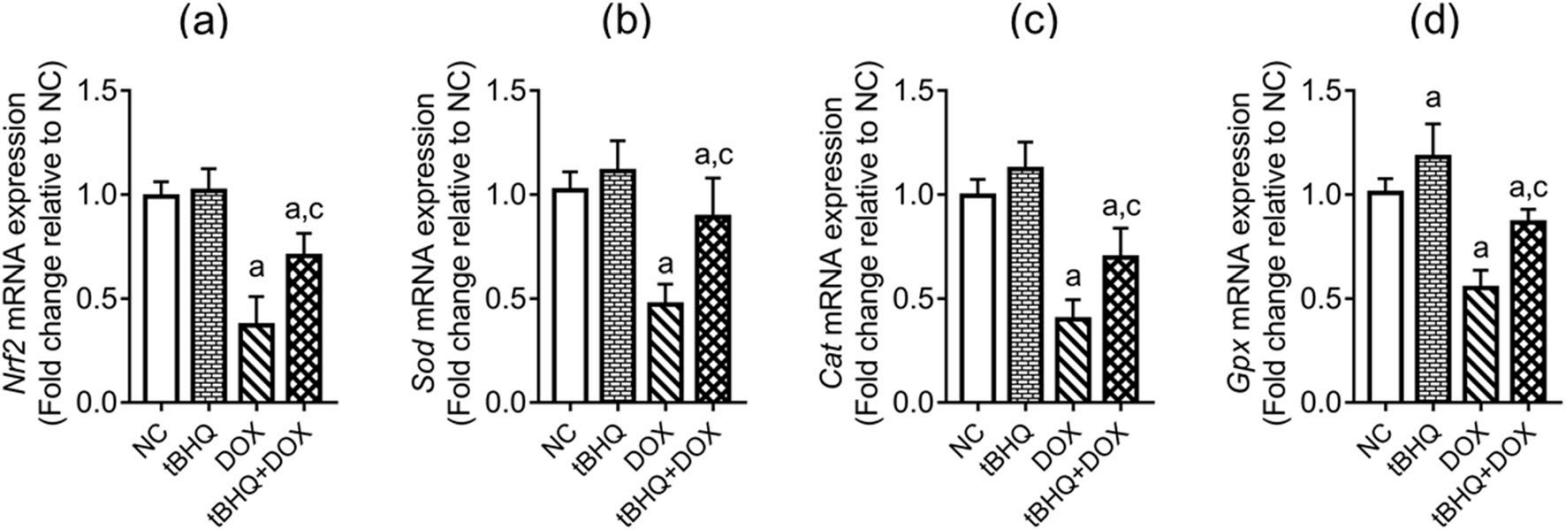
Effect of tBHQ on *Nrf2* (**a**), *Sod* (**b**), *Cat* (**c**) and *Gpx* (**d**) mRNA levels in the testis of DOX-treated rats. NC: normal control, tBHQ: tert-butylhydroquinone, DOX: doxorubicin, tBHQ + DOX: tert-butylhydroquinone + doxorubicin, Nrf2: nuclear factor erythroid 2-related factor-2, SOD: superoxide dismutase, CAT: catalase, GPx: glutathione peroxidase. Values are mean ± SD, n = 6. ^a^*p* < 0.05 versus NC; ^c^*p* < 0.05 versus DOX (One-way ANOVA with Tukey’s post-hoc test).

DOX downregulated (*p* < 0.05) the expression of *Nrf2*, glutathione peroxidase (*Gpx*), superoxide dismutase (*Sod*) and catalase (*Cat*) genes in the testis, compared to the NC group (Fig. 4a–d). tBHQ intervention, however, ameliorated the effects of DOX on these genes (Fig. 4a–d).

### Testicular antioxidant/oxidant parameters

The activities of antioxidant enzymes, notably: SOD, GPx, CAT and glutathione reductase (GR), and glutathione (GSH) level in the testes reduced (*p* < 0.05) in DOX group compared to the NC group (Table 3). tBHQ ameliorated the effect of DOX on SOD activity and prevented the changes in GPx, CAT and GR activities, and GSH level (Table 3). Furthermore, total antioxidant capacity (TAC) decreased (*p* < 0.05), while hydrogen peroxide (H_2_O_2_) and malondialdehyde (MDA) levels increased (*p* < 0.05) in the testis following DOX administration, when compared to the NC group (Table 3). However, tBHQ ameliorated DOX effects on these parameters (Table 3).

**Table 3:** Testicular antioxidant/oxidant parameters in the different experimental groups.

Parameter	NC	tBHQ	DOX	tBHQ + DOX
SOD activity (U/mg protein)	2.86 ± 0.34	2.91 ± 0.27	1.08 ± 0.22^a^	2.13 ± 0.31^a,c^
CAT activity (U/mg protein)	35.72 ± 3.24	38.76 ± 5.89	10.37 ± 1.42^a^	34.39 ± 3.16^c^
GPx activity (U/mg protein)	30.80 ± 2.92	31.35 ± 1.63	8.78 ± 0.68^a^	28.49 ± 3.23^c^
GR activity (U/mg protein)	21.05 ± 1.64	21.71 ± 1.77	8.22 ± 0.94^a^	19.33 ± 1.98^c^
GSH level (nmol/mg protein)	1.96 ± 0.16	2.18 ± 0.19	0.65 ± 0.13^a^	2.01 ± 0.10^c^
TAC (nmol/mg protein)	261.60 ± 17.56	266.00 ± 13.28	81.59 ± 8.17^a^	232.60 ± 22.04^a,c^
H_2_O_2_ level (µmol/mg protein)	2.01 ± 0.17	1.83 ± 0.21	4.23 ± 0.45^a^	2.59 ± 0.24^a,c^
MDA level (nmol/mg protein)	2.92 ± 0.24	2.47 ± 0.35	18.32 ± 1.02^a^	7.41 ± 0.63^a,c^

Values are mean ± SD, n = 6/group. NC: normal control, tBHQ: tert-butylhydroquinone, DOX: doxorubicin.

^a^*p* < 0.05 versus NC group, ^c^*p* < 0.05 versus DOX group (One-way ANOVA with Tukey’s post-hoc test).

### Cauda epididymal antioxidant/oxidant parameters

The activities of SOD, GPx, CAT and GR decreased (*p* < 0.05) in the cauda epididymis of rats in DOX group relative to the NC group, but these changes were ameliorated by tBHQ (Table 4). GSH level and TAC in the cauda epididymis were decreased (*p* < 0.05), while H_2_O_2_ and MDA levels increased (*p* < 0.05) following DOX administration, relative to the NC group (Table 4). Administration of tBHQ prevented these changes in the cauda epididymis (Table 4).

**Table 4:** Epididymal antioxidant/oxidant parameters in the different experimental groups.

Parameter	NC	tBHQ	DOX	tBHQ + DOX
SOD activity (U/mg protein)	2.62 ± 0.23	2.78 ± 0.13	1.26 ± 0.24^a^	2.18 ± 0.34^a,c^
CAT activity (U/mg protein)	27.31 ± 2.17	27.92 ± 3.32	12.04 ± 1.67^a^	22.29 ± 2.07^a,c^
GPx activity (U/mg protein)	28.59 ± 1.45	30.67 ± 1.42	10.07 ± 0.98^a^	23.84 ± 3.62^a,c^
GR activity (U/mg protein)	18.85 ± 1.07	19.86 ± 0.82	8.00 ± 0.77^a^	15.24 ± 1.93^a,c^
GSH level (nmol/mg protein)	1.89 ± 0.34	2.02 ± 0.36	0.68 ± 0.14^a^	1.57 ± 0.28^c^
TAC (nmol/mg protein)	214.40 ± 13.60	223.60 ± 9.90	82.82 ± 8.19^a^	197.40 ± 23.72^c^
H_2_O_2_ level (µmol/mg protein)	1.73 ± 0.14	1.65 ± 0.19	3.34 ± 0.21^a^	1.91 ± 0.38^c^
MDA level (nmol/mg protein)	1.50 ± 0.11	1.43 ± 0.11	7.06 ± 0.75^a^	2.03 ± 0.33^c^

Values are mean ± SD, n = 6/group. NC: normal control, tBHQ: tert-butylhydroquinone, DOX: doxorubicin.

^a^*p* < 0.05 versus NC group, ^c^*p* < 0.05 versus DOX group (One-way ANOVA with Tukey’s post-hoc test).

### Expression of inflammation-related genes in the testis

**Figure 5:**
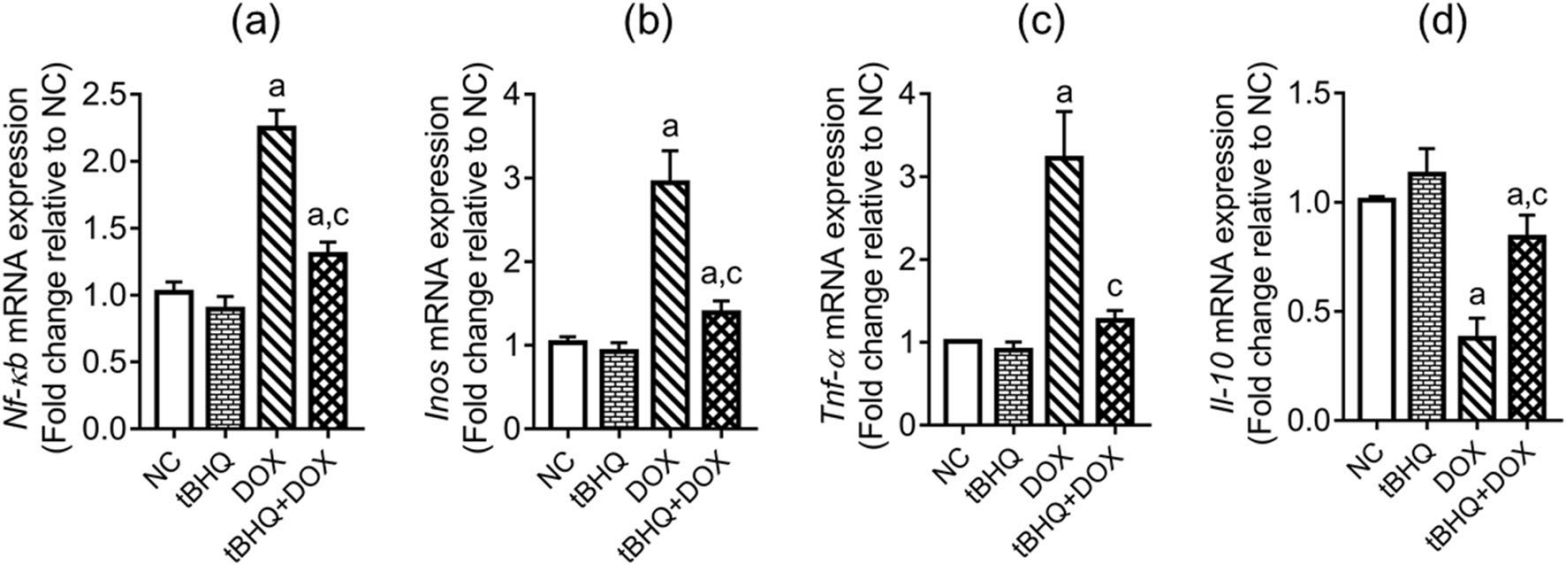
Effect of tBHQ on *Nf-κb* (**a**), *Inos* (**b**), *Tnf-α* (**c**) and *Il-10* (**d**) mRNA levels in the testis of DOX-treated rats. NC: normal control, tBHQ: tert-butylhydroquinone, DOX: doxorubicin, tBHQ + DOX: tert-butylhydroquinone + doxorubicin, *Nf-κb*: nuclear factor kappa B, *Inos*: inducible nitric oxide synthase, *Il*: interleukin, *Tnf-α*: tumor necrosis factor alpha. Values are mean ± SD, n = 6/group. ^a^*p* < 0.05 versus NC; ^c^*p* < 0.05 versus DOX (One-way ANOVA with Tukey’s post-hoc test).

The expression of pro-inflammatory genes, notably; *Nf-κb*,* Tnf-α* and *Inos* were upregulated (*p* < 0.05) while the expression of the anti-inflammatory *Il-10* gene was downregulated (*p* < 0.05) in the testis of rats in DOX group when compared to the NC group (Fig. 5a–d). However, tBHQ ameliorated DOX effects on *Nf-κb*, *Inos* and *Il-10* genes, and prevented the change in *Tnf-α* gene expression (Fig. 5a–d).

### Expression of apoptosis-related genes in the testis

**Figure 6:**
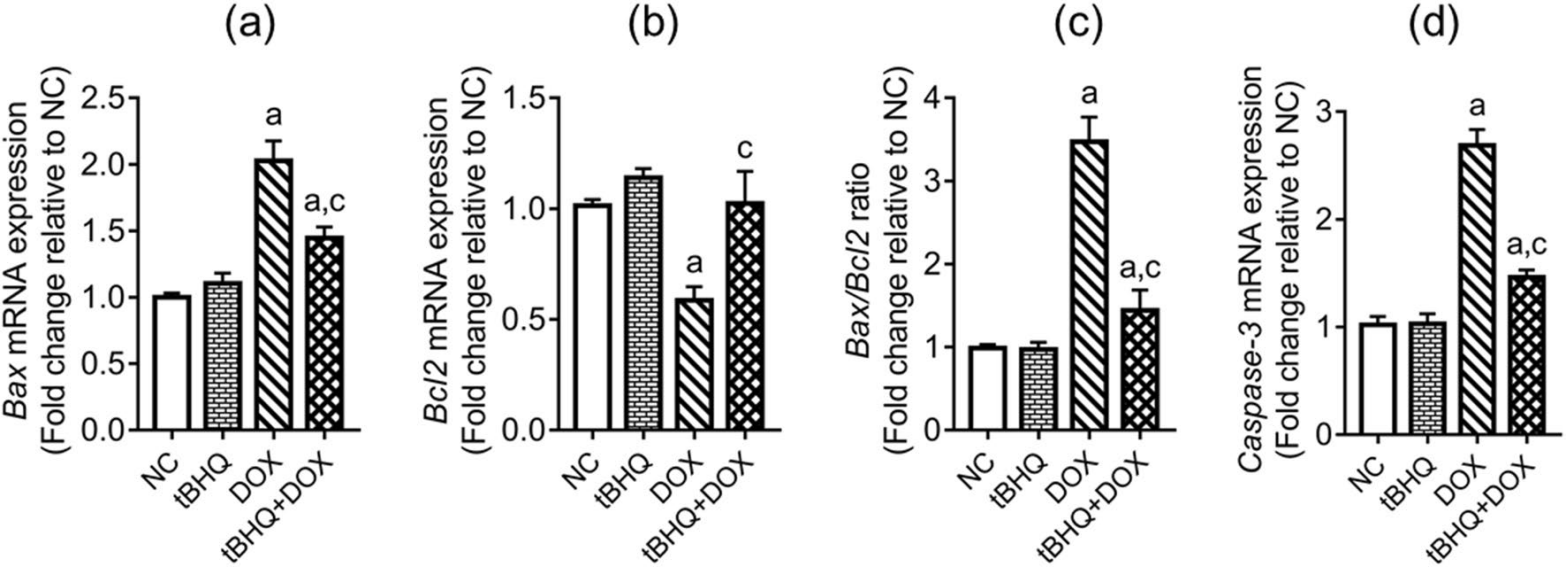
Effect of tBHQ on *Bax* (**a**), *Bcl2* (**b**), *Bax/Bcl2* ratio (**c**) and *Caspase-3* (**d**) mRNA levels in the testis of DOX-treated rats. NC: normal control, tBHQ: tert-butylhydroquinone, DOX: doxorubicin, tBHQ + DOX: tert-butylhydroquinone + doxorubicin,* Bcl-2*: Beta cell lymphoma-2, *Bax*: Bcl-2-associated X protein. Values are mean ± SD, n = 6. ^a^*p* < 0.05 versus NC; ^c^*p* < 0.05 versus DOX (One-way ANOVA with Tukey’s post-hoc test).

DOX upregulated (*p* < 0.05) the testicular expression of pro-apoptotic (*Bax* and *Caspase-3*) genes, and downregulated (*p* < 0.05) the anti-apoptotic *Bcl2* gene expression, when compared to the NC group (Fig. 6a–d). Furthermore, the ratio of *Bax/Bcl2* increased in DOX group, relative to the NC group (Fig. 6c). These DOX-induced changes were ameliorated by tBHQ (Fig. 6a–d).

### Protein levels of cleaved caspase-3 and proliferating cell nuclear antigen (PCNA) in the testis

**Figure 7:**
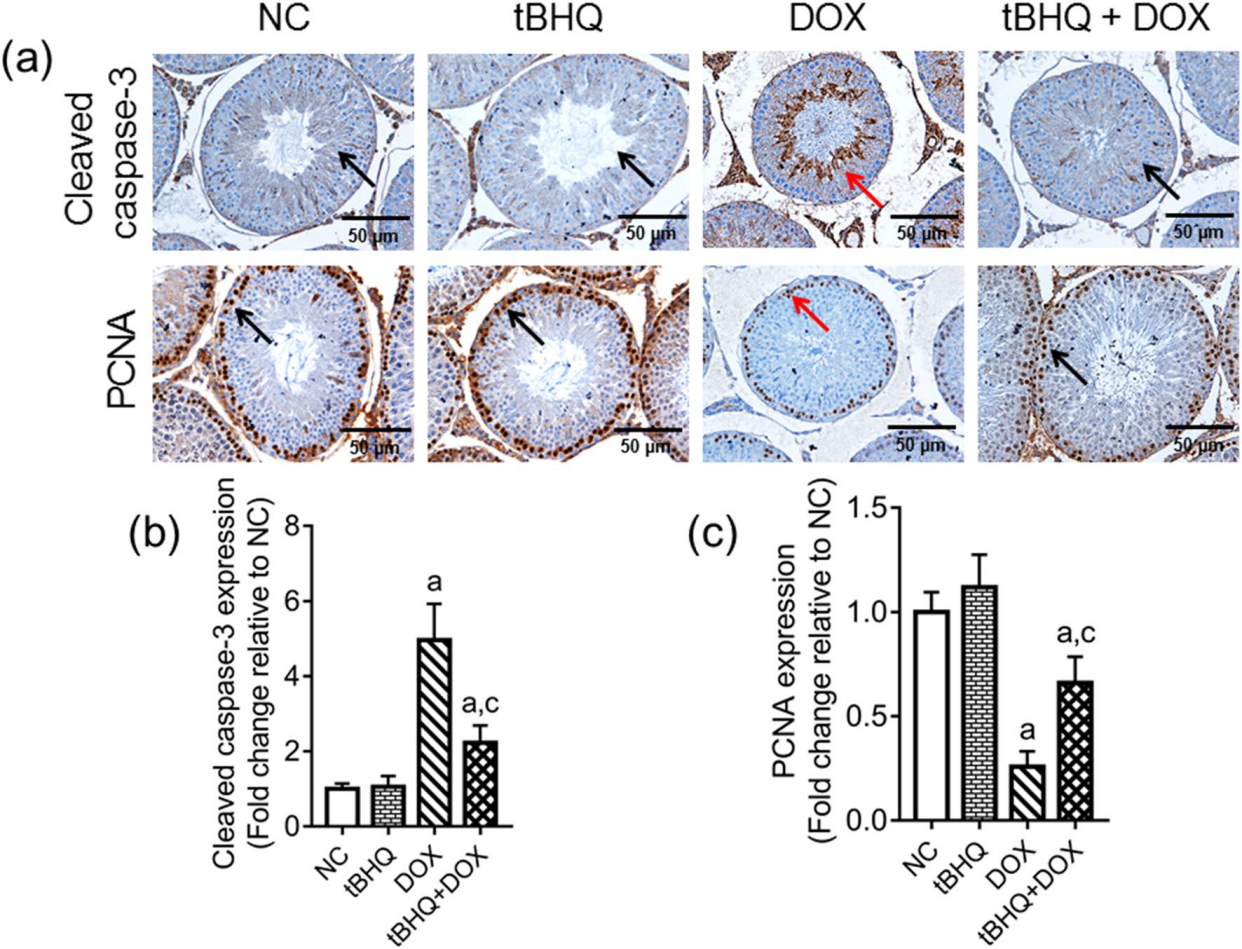
Effect of tBHQ on cleaved caspase-3 protein level (**a**,**b**) and PCNA protein level (**a**,**c**) in the testis of DOX-treated rats. NC: normal control, tBHQ: tert-butylhydroquinone, DOX: doxorubicin, PCNA: proliferating cell nuclear antigen. Cleaved caspase-3 protein level was highest in the seminiferous tubules of DOX group (red arrow) compared with the other three groups (black arrow), while PCNA protein level was lowest in the seminiferous tubules of DOX group (red arrow) relative to the other three groups (black arrow). Magnification: × 400, scale bar: 50 µm. For numerical data representing cleaved caspase-3 (**b**) and PCNA (**c**) protein levels, values are mean ± SD, n = 6/group. ^a^ *p* < 0.05 versus NC; ^c^ *p* < 0.05 versus DOX (One-way ANOVA with Tukey’s post-hoc test).

The level of cleaved caspase-3 protein was increased in the testes of rats in DOX group as demonstrated by the increased brown staining in the representative photograph (Fig. 7a). The corresponding quantitative data showed increased staining (*p* < 0.05) in DOX group relative to the NC group, while tBHQ ameliorated this effect (Fig. 7b). Conversely, the level of PCNA protein (a proliferation marker) was decreased in the testes of rats in DOX group as demonstrated by the decreased brown staining in the representative photograph (Fig. 7a). Quantitative data showed that PCNA protein in the testis decreased (*p* < 0.05) in DOX group when compared to the NC group, while tBHQ ameliorated this effect (Fig. 7c, d).

## Discussion

**Figure 8:**
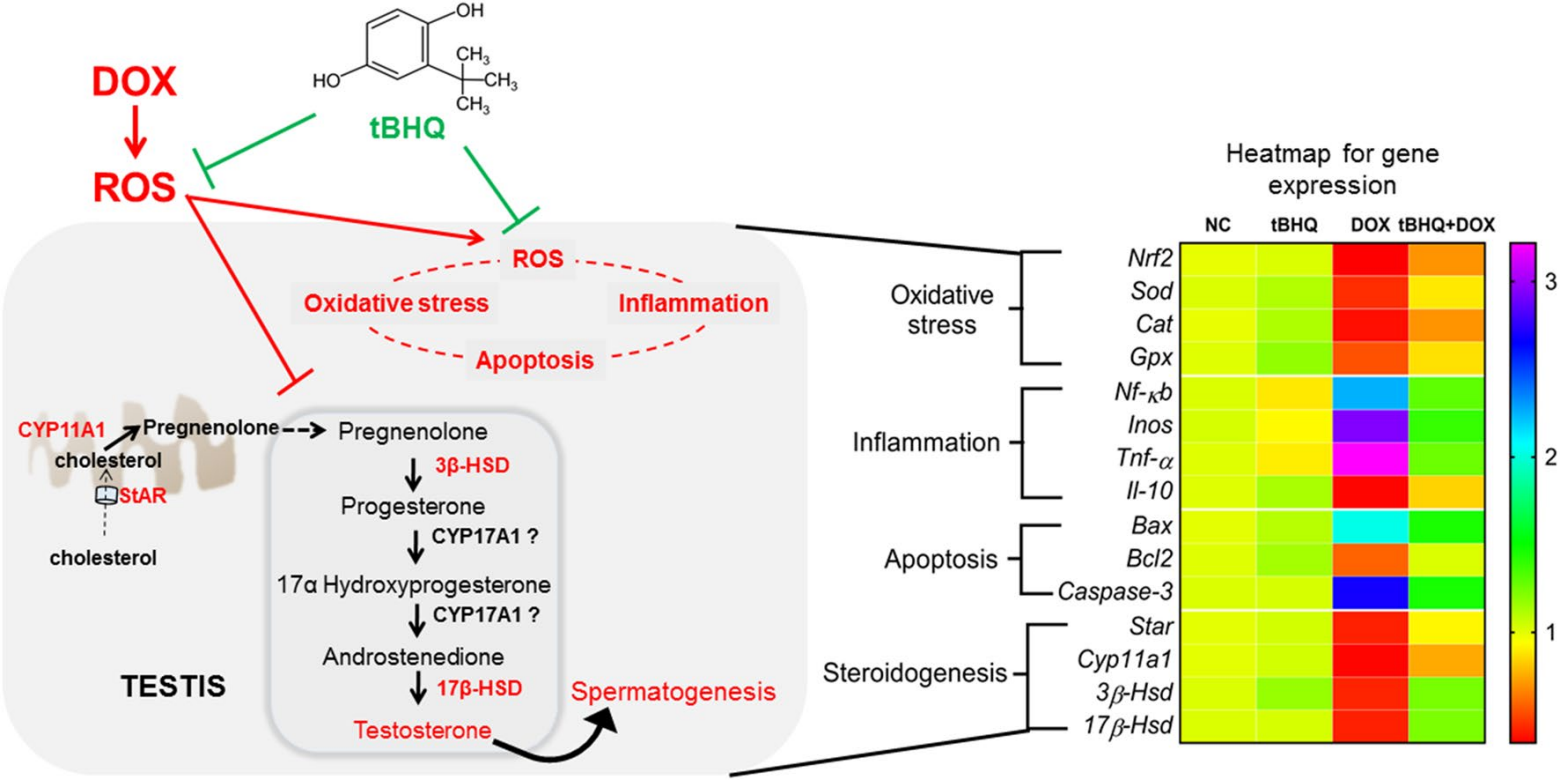
Schematic representation of the effect of tBHQ on DOX-induced testicular toxicity. DOX triggered ROS generation, causing oxidative stress, which in turn activated cytotoxic genes that control inflammation and apoptosis. Also, DOX-induced ROS generation altered genes that regulate steroidogenesis, causing testosterone decline and spermatogenesis impairment. However, pre-treatment with tBHQ offered some level of protection against DOX-induced toxicity as demonstrated by the heatmap of affected genes which have been grouped according to their targeted pathways. The heatmap key on the right side shows the expression levels of the genes; numbers greater than 1 depict upregulation while numbers below 1 depict downregulation. NC: normal control, tBHQ: tert-butylhydroquinone, DOX: doxorubicin, tBHQ + DOX: tert-butylhydroquinone + doxorubicin.

DOX has been in use for several decades and is considered as a highly potent and effective chemotherapeutic drug. It is, however, associated with male infertility as it also targets healthy cells in the testis, killing them. This toxic effect is mediated by ROS^8^, with its accompanying peroxidation of the lipid membranes of germ cells and spermatozoa, inflammation and apoptosis^36^. This informed the choice of the antioxidant, tBHQ, to investigate the likely protection against DOX-mediated male reproductive toxicity. Herein, we have demonstrated that tBHQ reduces the negative effects of DOX on the testis by targeting oxidative stress, inflammation and apoptosis, and suppressing its negative effects on steroidogenesis, thus, improving spermatogenesis (Fig. 8).

Our results showed that DOX administration induced weight loss in the present study, consistent with previous reports^37,38^. DOX is reported to decrease glucose uptake by skeletal muscles leading to weight loss^39^, thus, corroborating our observed result. Furthermore, DOX triggered oxidative stress in the testis and cauda epididymis, which is supported by other reports that DOX generates ROS with a resultant increase in lipid peroxidation^14,16,40^. Several studies have shown that tBHQ protects against oxidative damage by activating Nrf2, which increases the expression of ARE genes^26,29,41,42^. Although we did not observe *Nrf2* gene upregulation in normal rats administered tBHQ in the present study, we found that tBHQ, to some extent, could reduce DOX-induced downregulation of *Nrf2* gene expression in tBHQ + DOX administered rats. While we did not assay for all the members of the ARE genes, we observed that tBHQ reduced DOX-induced changes in the expression of some cytoprotective genes (*Sod*, *Gpx* and *Cat*; Fig. 8), which likely increased the activities of antioxidant enzymes (SOD, CAT, GPx and GR) and decreased H_2_O_2_ and lipid peroxidation as demonstrated by the decreased MDA level. This is thus, a protective mechanism of tBHQ against DOX-induced oxidative injury, and is consistent with previous reports of its protective effects against oxidative stress induced by arsenic^43^, gastric ulcer^44^, DOX^35^ and cisplatin^29^.

Oxidative stress is most often accompanied by inflammation, since ROS can activate pro-inflammatory transcription factors^45,46^. The upregulation of the pro-inflammatory transcription factor *Nf-κb*,* Inos* and *Tnf-α* shown here, agrees with previous investigations demonstrating that DOX upregulated NF-κB and increased pro-inflammatory markers; iNOS, TNF-α and IL-1β, in the heart^45^ and testis^17^. In the present study, tBHQ attenuated DOX-induced testicular inflammation, thus, corroborating a previous study showing that tBHQ decreased inflammatory activity in gut cells as demonstrated by decreases in the levels of NF-κB, TNF-α, IL-1β and IL-6^30^.

ROS is one of the culprits that triggers the apoptotic pathway^47^. In a study investigating the influence of DOX on apoptosis in breast cancer cell lines, DOX upregulated Bax, caspase-8 and caspase-3 expressions while downregulating the anti-apoptotic Bcl2 and Bcl-xL levels^48^. However, DOX has also been reported to induce apoptosis in non-cancerous target cells as demonstrated by Bax and caspase-3 upregulation^16,49^. These are consistent with our findings (Fig. 8). We assessed the level of cleaved caspase-3 protein in the testis and found a significant increase in DOX group relative to the NC group, suggesting an increase in apoptosis. Although the testis contains different cell types, the germ cells form the bulk of the cell types. Therefore, we hypothesize that the increased cleaved caspase-3 level in the testis of rats in DOX group may be indicative of an increase in the apoptotic rate of germ cells. Similarly, the decreased PCNA protein level in the DOX group may suggest poor proliferating capacity of the germ cells, which is detrimental to spermatogenesis. This hypothesis is drawn from the fact that PCNA weakly stains non-proliferating cells, and in the context of the present study, Sertoli cells^50^. Furthermore, proliferating germ cells outnumber the Sertoli cells in the basal area of the seminiferous tubule. Therefore, most of the PCNA-stained cells in the present study are likely to be proliferating germ cells. Pre-treatment with tBHQ attenuated DOX-mediated apoptosis in the present study, and may involve decreased Nrf2 downregulation, since Nrf2 has been reported to decrease apoptosis by activating cytoprotective proteins including Bcl2^51,52^.

The cytotoxic effects of DOX may have induced adverse effects on testicular tissues that are capable of impairing fertility, as shown by dysregulation of steroidogenesis and impairment of spermatogenesis. The former could have resulted from the downregulation of *Star*,* Cyp11a1*,* 3β-Hsd* and *17β-Hsd* genes and their respective protein levels. The transport of cholesterol by StAR from the outer mitochondria membrane into the inner mitochondria membrane for the first enzymatic step in steroidogenesis, involving CYP11A1, serves as the rate-limiting step for steroid hormone synthesis^53^. Therefore, the decreased levels of *Star* and *Cyp11a1* genes in DOX group in the present study may suggest that this all-important rate-limiting step may have been impaired, as supported by the observed decrease in testosterone level (Fig. 8). Besides the decreased expression of steroidogenic genes and testosterone level, we found that LH level and Leydig (steroidogenic) cell count also decreased following DOX therapy. The decrease in Leydig cell count may have been due to apoptosis, and the decrease in LH level despite the low testosterone level in DOX group may suggest a defective negative feedback mechanism since testosterone production is regulated by LH release. Our observations are similar to those from two separate studies that reported the downregulation of steroidogenic genes in male rats following DOX therapy^16,21^.

Considering the role of hormones, notably LH, FSH and testosterone, in maintaining spermatogenesis, we hypothesize that the decline in the levels of these hormones in the DOX group may have contributed to spermatogenesis impairment (decrease in sperm count) and sperm abnormalities in the present study, as well as germ cell apoptosis. Also, the decreased seminiferous tubular diameter, epithelial height, and relative testes and epididymal weights support our hypothesized spermatogenesis impairment. Our findings on sperm abnormalities are consistent with previous reports on animal models of DOX-induced testicular toxicity^16,21^, and reports from a clinical study of men on DOX therapy^54^. Pre-intervention with tBHQ reduced the negative effects of DOX on testicular steroidogenesis and spermatogenesis in the present study.

## Conclusion

Pre-treatment of DOX-administered rats with tBHQ ameliorates testicular toxicity by dampening DOX-induced dysregulation of cytoprotective genes, thus, supressing oxidative stress, inflammation and apoptosis. These beneficial effects of tBHQ may be responsible for the improvements in steroidogenesis and spermatogenesis (Fig. 8). Further studies are required to assess the clinical application of tBHQ and its protective effect on the reproductive health of patients undergoing chemotherapy.

## Materials and methods

### Chemicals

Doxorubicin was bought from Khandelwal Laboratories Pvt Ltd (Mumbai, India). tBHQ was obtained from International Laboratory (USA). RNA extraction kit was obtained from Analytik Jena AG (Jena, Germany), RNAlater was bought from Sigma Aldrich (St. Louis, MO, USA). SensiFAST SYBR Hi-ROX One-Step PCR kit was bought from Bioline (UK) with primers synthesized by Integrated DNA Technologies (Malaysia). Rabbit polyclonal primary antibodies for caspase-3 (PAA626Ra01) and PCNA (PAA591Mi01) were obtained from Cloud-Clone Corp (Katy, TX, USA). Dako EnVision^+^ System/HRP-labelled polymer containing goat anti-rabbit secondary antibody (K4003) was purchased from Agilent Technologies, Inc. (Santa Clara, CA, USA). All other chemicals were of analytical grade.

### Experimental animals

Twenty-four sexually mature male Albino Wistar rats (12 weeks old) weighing 220–240 g were purchased from the Animal House of the Department of Physiology, University of Calabar, Calabar, Nigeria. The animals were housed in the Department of Biochemistry Animal House. They were acclimatized for seven days in plastic cages with wire mesh and the cages were provided with absorbent beddings. The animals were allowed to feed freely on standard rat pellet and water during this period. The animals were exposed to 12/12 h light and dark cycles. The study was granted approval by the Animal Ethics Committee of the Faculty of Basic Medical Sciences (82PHY10220). Furthermore, all experiments performed in this study were in accordance with the principles of care and use of experimental animals as prescribed by the National Institute of Health and conforms to the ARRIVE guidelines.

### Experimental design

Twenty-four male rats were randomly assigned into four groups consisting of six animals each (n = 6). Group 1 served as normal control (NC) and received oral DMSO (1%) for 14 days with normal saline by intraperitoneal (i.p) injection on the 8th day of the study. Group 2 (tBHQ) received orally, 50 mg/kg body weight (b.w.) of tBHQ in 1% DMSO for 14 days with intraperitoneal injection of normal saline on the 8th day. Group 3 (DOX) received i.p. injection of DOX (7 mg/kg b.w.) on the 8th day and oral 1% DMSO for 14 days. Group 4 (tBHQ + DOX) received both tBHQ and DOX at doses described for groups 2 and 3, respectively. After the last tBHQ dose on day 14, the rats were fasted overnight and sacrificed on the 15th day. The dose of DOX used in this study was reported to induce oxidative stress and nephrotoxicity in rats^55^, and is equivalent to 42 mg/m^2^ human dose, which is within the often prescribed clinically effective dose range of 40–75 mg/m^2^ every 3–4 weeks^56^. On the other hand, the dose of tBHQ used in the present study has been reported to activate cytoprotective genes in rat models of oxidative injury^29,57^.

### Tissue collection and preparation

The animals were weighed for final body weight on the 15th day before they were euthanized using pentobarbital (60 mg/kg b.w.)^45^. Epididymis and testes were excised, rinsed in phosphate buffered saline (PBS), pH 7.4, pad-dried and weighed. The vas deferens was harvested for sperm analysis. Part of the harvested right testis was placed in RNAlater and preserved at − 80 °C for gene expression studies. The other part of the right testis together with the corresponding cauda epididymis were separately homogenized in ice-cold PBS, pH 7.4. The homogenate was centrifuged for 20 min using a refrigerated centrifuge at 1000 × *g*. The resulting supernatant was retrieved and stored at − 80 °C until needed.

### Sperm analyses

The analyses of sperm count, motility, morphology and viability were done using previously described methods^58,59^. Briefly, the left vas deferens was placed in 1 mL of normal saline and was gently massaged for the sperm cells to swim into the saline. Thereafter, the mixture was gently stirred. One drop of the sperm suspension was immediately placed on the mounting stage of a Markler’s counting chamber (Sefi-Medical Instruments, Israel) and viewed under a light microscope (Olympus BX41, Olympus Corporation, Tokyo, Japan). The number of motile spermatozoa was counted in 10 random fields and the result was expressed as a percentage of the total number of spermatozoa in the same fields. This procedure was performed in duplicates for each sample.

Epididymal sperm count was assessed following a previously described method^58^. Briefly, the left cauda epididymis was shredded in 2 mL of normal saline and filtered with a nylon mesh. The filtrate (sperm suspension) was stained with eosin-Y solution. The stained sperm suspension was then aliquoted using leucocyte haemocytometer (up to the 0.5 mark) and diluted with normal saline (up to the 11 mark). The suspension was mixed, and few drops were discarded before charging into both chambers of a Neubauer hemocytometer. Sperm heads were counted in 5 squares of each chamber of the hemocytometer under a light microscope using routine counting techniques. The average value from both chambers was recorded as sperm count.

Epididymal sperm viability was assessed as previously reported^59^. Stained sperm suspension was smeared on a glass slide, covered with a cover slip, and viewed under a light microscope using 100 × objective. The number of viable spermatozoa (unstained sperm heads) were counted in 200 spermatozoa and expressed as a percentage of the total spermatozoa counted.

To assess sperm abnormal morphology, sperm smear was examined for the presence of spermatozoa abnormalities such as head and tail defects. For each slide, 200 spermatozoa were counted, and the number of defective spermatozoa was expressed as a percentage of the total number of spermatozoa counted^59^.

### Hormonal and steroidogenesis marker proteins assays

Testicular homogenates were assessed for testosterone (AA E-1400), FSH (QY-E11462) and LH (QY-E11465) using ELISA kits (Labor Diagnostika Nord GmbH, Germany for testosterone, Qayee Bio-technology, Shangai, China, for LH and FSH) following the manufacturers’ protocols. StAR (QY-E11839), CYP11A1 (QY-E11837), 3β-HSD (QY-E11838) and 17β-HSD (QY-E11840) protein levels in the testes were assayed using ELISA kits (Qayee Bio-technology, Shangai, China) following the manufacturer’s protocols. For all the kits, the intra-assay coefficient of variation was < 10%.

### Antioxidant/oxidant assay in the testes and epididymis

Nitro tetrazolium blue reduction assay was used to determine SOD activity in testicular and epididymal homogenates^60^. GPx activity was assayed using the principle of oxidation of GSH by H_2_O_2_^61^. CAT activity level was assayed on the principle of molybdate reaction with hydrogen peroxide as described by Goth^62^. The activity of GR was determined using method described by Carlberg and Mannervik^63^. GSH level were assessed according to the method previously described by Jollow et al.^64^. TAC was evaluated using the method of Koracevic et al.^65^, while MDA level was measured according to the method of Ohkawa et al.^66^. These methods are provided in detail in the supplementary file. H_2_O_2_ (DIOX-250) and total protein (QCPR-500) levels were assayed using colorimetric assay kits (BioAssay Systems, California, USA).

### Histopathology of testis

The left testis was stored for 18–24 h in Bouin’s fixative. It was dehydrated and then embedded in paraffin blocks. Sections of 5 µm were obtained and stained with haematoxylin and eosin (H&E) technique. Using a light microscope (Olympus BX41, Olympus Corporation, Tokyo, Japan), 200 seminiferous tubules were evaluated for germ cell loss (either focal or generalized) and results were expressed as a percentage of the total number of tubules examined. The diameter and epithelial height of 20 randomly selected seminiferous tubules were measured at × 100 magnification using Image Analyser software. Leydig cells were also counted in 20 randomly selected inter-tubular spaces^45,59^. The method described by Johnsen^67^ was used to assess Johnsen testicular biopsy score in 10 tubules selected at random using the scoring criteria in Supplementary Table 1. For all the parameters, quantitative data were collected from three separate sections per animal and the average for each animal was calculated.

### Regulatory genes expression for steroidogenesis, antioxidants, inflammation and apoptosis

RNA extraction was performed on 20–30 mg of testicular tissue using a total RNA extraction kit (Analytik Jena, Germany) following the protocol by manufacturer. Agarose gel (1% w/v) electrophoresis was used to assess RNA integrity and viewed using a UV transilluminator (ChemiDoc XRS, Bio-Rad Laboratories, Hercules, CA, USA), while Nanodrop spectrophotometer (Eppendorf Nanodrop BioPhotometer plus, Hamburg, Germany) was used to assess the concentration and purity of extracted RNA. Samples with distinct 28S and 18S ribosomal RNA bands and showed OD_260/280_ of 1.8–2.0 were considered pure and used for the downstream experiments.

SensiFAST SYBR Hi-Rox One-Step PCR kit (Bioline, London, UK) with StepOnePlus Real-Time PCR system (Applied Biosystems Co. Foster City, CA, USA) was used to perform Real-time RT-qPCR following the manufacturer’s guidelines. From previous studies, primer sequences of target genes were selected and confirmed from GenBank (Supplementary Table 2). These primer pairs have been used in our previous experiments^45,59,68^ and their specificities were confirmed using melting curve and their efficiencies were confirmed to be 90–100%. For the comparative experiments, after an initial denaturation for 2 min at 95 °C, three-step cycling was performed, and involved denaturation for 5 s at 95 °C, annealing at 60 °C for 10 s, and extension at 72 °C for 5 s. A total of 40 cycles were performed. Livak’s method (2^−ΔΔCt^)^69^ was used for relative gene expression and results were normalised to *Gapdh* which was used as the house keeping gene since the expression level did not change across the samples. All mRNA expression experiments were performed in triplicates.

### Immunohistochemistry for assay of cleaved caspase-3 and PCNA staining

Testicular sections of 5 µm were used for caspase-3 and PCNA immunostaining. Tris–EDTA buffer with 0.05% tween 20, pH 9.0 was used for antigen retrieval in a pressure cooker for 5 min. Thereafter, endogenous peroxidase blocking was performed using 3% H_2_O_2_ in PBS for 5 min. Sections were incubated overnight at 4 °C with rabbit polyclonal primary antibodies for caspase-3 (PAA626Ra01, 1:150) and PCNA (PAA591Mi01, 1:50) (Cloud-Clone Corp, Houston, TX, USA) after rinsing with distilled water and tris-buffered saline containing 0.05% tween 20 (TBST, pH 8.4). Sections were rinsed twice for 5 min with TBST and incubated at room temperature for 30 min using Dako EnVision + System/HRP-labelled polymer containing goat anti-rabbit secondary antibody (catalogue number: K4003, Agilent Technologies, Inc. Santa Clara, USA). DAB substrate (Agilent Technologies, Inc. Santa Clara, USA) was then utilised for detection. Sections were counter-stained with haematoxylin and viewed under a light microscope (Olympus BX41, Olympus Corporation, Tokyo, Japan). ImageJ software (NIH-Bethesda, MD, USA) was used to analyse the intensity of staining and percentage positive cells, for both caspase-3 and PCNA proteins. The results were expressed as fold change relative to the normal control group.

### Statistical analysis

Results are expressed as mean ± standard deviation (SD). Graph Pad Prism 7.0 (Graph Pad Software Inc., La Jolla, CA, USA) was used to analyse datasets. Shapiro–Wilk and D’Agostino–Pearson Omnibus normality tests were used to confirm that all data were normally distributed. Consequently, one-way analysis of variance (ANOVA) and Tukey’s post-hoc test was used to determine differences between groups. *P* < 0.05 was used to determine statistical significance.

## Supplementary Information

Supplementary information.

## Data Availability

The data used for this study are available from the corresponding author upon request.
