# Supplementary material for: Tert-butylhydroquinone attenuates doxorubicin-induced dysregulation of testicular cytoprotective and steroidogenic genes, and improves spermatogenesis in rats
Source: Sci Rep. 2021 Mar 9;11:5522. doi: 10.1038/s41598-021-85026-7 (PMC7970903; doi:10.1038/s41598-021-85026-7)
Supplement: Supplementary file 1 — Supplementary information. [file 41598_2021_85026_MOESM1_ESM.pdf]

Tert-butylhydroquinone attenuates doxorubicin-induced dysregulation of testicular cytoprotective and steroidogenic genes, and improves spermatogenesis in rats

Godwin Adakole Ujah[1], Victor Udo Nna[1*], Joseph Bagi Suleiman[2,3], Chinedum Eleazu[2,4], Chukwuemeka Nwokocha[5], Joy Assima Rebene[1], Michael Umana Imowo[1], Emmanuel Ochui Obi[1], Charlette Amachree[1], Evarest Chigozie Udechukwu[1], Mahaneem Mohamed[2,6*]

[1] Department of Physiology, College of Medical Sciences, University of Calabar, P.M.B. 1115 Calabar, Cross River State, Nigeria.
[2] Department of Physiology, School of Medical Sciences, Universiti Sains Malaysia, 16150 Kubang Kerian, Kelantan, Malaysia.
[3] Department of Science Laboratory Technology, Akanu Ibiam Federal Polytechnic, Unwana, Afikpo, Ebonyi State, Nigeria.
[4] Department of Chemistry, Biochemistry and Molecular Biology, Alex Ekwueme Federal University, Ndufu-Alike, Ikwo, Ebonyi State, Nigeria.
[5] Department of Basic Medical Sciences (Physiology Section). The University of the West Indies, Mona, Kingston 7, Jamaica.
[6] Unit of Integrative Medicine, School of Medical Sciences, Universiti Sains Malaysia, 16150 Kubang Kerian, Kelantan, Malaysia.

Corresponding Authors:

Mahaneem Mohamed (MD, MSc, PhD)

Department of Physiology, School of Medical Sciences, Universiti Sains Malaysia, 16150 Kubang Kerian, Kelantan, Malaysia. mahaneem@usm.my

Victor Udo Nna (BSc, MSc, PhD)

Department of Physiology, College of Medical Sciences, University of Calabar, P.M.B. 1115 Calabar, Cross River State, Nigeria. victorudon@unical.edu.ng

Supplementary Table 1. Johnsen score (Johnsen, 1970)

Score	Stage of spermatogenesis
10	Full spermatogenesis
9	Disorganised tubular epithelium with many late spermatids.
8	Few late spermatids.
7	Many early spermatids; no late spermatids.
6	No late spermatids; arrest of spermatogenesis at the spermatid stage.
5	Many spermatocytes; no spermatids.
4	Arrest of spermatogenesis at the primary spermatocyte stage; no spermatids.
3	Only spermatogonia.
2	Sertoli cells only; no germ cells.
1	Tubular sclerosis; absence of seminiferous epithelial cells.

Supplementary Table 2. Primer sequence for PCR amplification of regulatory genes for steroidogenesis, oxidative stress, inflammation and apoptosis.

Target gene	Accession number	Primer sequence (5' – 3')		Size (bp)
Star	NM_031558.3	^{F}GGGCATACTCAACAACCAG	^{R}ACCTCCAGTCGGAACACC	111
Cyp11a1	J05156	^{F}CTTTGGTGCAGGTGGCTAG	^{R}CGGAAGTGCGTGGTGTTT	115
3β-Hsd	M38178	^{F}TGTGCCAGCCTTCATCTAC	^{R}CTTCTCGGCCATCCTTTT	145
17β-Hsd	NM_054007	^{F}GACCGCCGATGAGTTTGT	^{R}TTTGGGTGGTGCTGCTGT	140
Nrf2	NM_031789.1	^{F}CAGGTTGCCCACATTCCCAA	^{R}ATATCCAGGGCAAGCGACTCAT	110
Sod	X05634.1	^{F}CGAGCATGGGTTCCATGTC	^{R}CTGGACCGCCATGTTTCTTAG	101
Cat	NM_012520.2	^{F}ACAACTCCCAGAAGCCTAAGAATG	^{R}GCTTTTCCCTTGGCAGCTATG	76
Gpx	NM_030826.4	^{F}GGAGAATGGCAAGAATGAAGA	^{R}CCGCAGGAAGGTAAAGAG	139
Nf-κb	NM_199267.2	^{F}CGCGGGGACTATGACTTGAA	^{R}AGTTCCGGTTTACTCGGCAG	163
Inos	XM_006246949.3	^{F}CAGCCCTCAGAGTACAACGAT	^{R}CAGCAGGCACACGCAATGAT	91
Tnf-α	NM_012675.3	^{F}ACTGAACTTCGGGGTGATCG	^{R}GCTTGGTGGTTTGCTACGAC	153
Il-10	NM_012854.2	^{F}TTGAACCACCCGGCATCTAC	^{R}CCAAGGAGTTGCTCCCGTTA	91
Bax	U49729.1	^{F}CGCGTGGTTGCCCTCTTCTACTTT	^{R}CAAGCAGCCGCTCACGGAGGA	124
Bcl-2	NM_016993.1	^{F}ATCGCTCTGTGGATGACTGAGTAC	^{R}AGAGACAGCCAGGAGAAATCAAAC	134
Caspase-3	NM_012922	^{F}AAGATACCAGTGGAGGCCGACTTC	^{R}GGGAGAAGGACTCAAATTCCGTGG	199
Gapdh	NM_017008	^{F}TCACCACCATGGAGAAGGC	^{R}GCTAAGCAGTTGGTGGTGCA	169

Bcl-2: Beta cell lymphoma-2, *Bax*: Bcl-2-associated X protein, *Caspase*: cysteine-aspartic proteases, *Cat*: catalase, *Cyp11a1*: cholesterol side chain cleavage enzyme, *Gapdh*: glyceraldehyde-3-phosphate dehydrogenase, *Gpx*: glutathione peroxidase, *Hsd*: hydroxysteroid dehydrogenase, *Il*: interleukin, *Inos*: inducible nitric oxide synthase, *Nf-κb*: nuclear factor kappa B, *Nrf2*: nuclear factor erythroid 2-related factor-2, *Sod*: superoxide dismutase, *Star*: steroidogenic acute regulatory protein, *Tnf*: tumor necrosis factor.

Antioxidant/oxidant assay in the testes and epididymis

Measurement of superoxide dismutase (SOD) activity

SOD activity was assessed using a method based on nitro tetrazolium blue (NBT) reduction [1]. A substrate mixture was first prepared by pipetting 27 mL of 50 mM phosphate buffer (pH 7.8), L-methionine solution (1.5 mL, 30 mg/mL), NBT.2HCl (1 mL, 1.41 mg/mL) and Triton X (0.75 mL, 1 %) into a dark bottle. The mixture was stirred and protected from light. Thereafter, 1 mL of substrate mixture was pipetted into a glass test tube that was wrapped with aluminium foil, followed by 20 μL of sample (or phosphate buffer for blank) and 10 μL of riboflavin solution. The mixture was placed in an aluminium box and irradiated with fluorescent lamps of 18 watts for 7 min, after which the absorbance of the mixture was read at 560 nm using a spectrophotometer. SOD activity was expressed as unit mg^{-1} protein, where 1 unit of enzyme was defined as the amount of enzyme required to reduce NBT by 50 %.

Measurement of catalase (CAT) activity

CAT activity was assessed based on enzyme-catalysed decomposition of hydrogen peroxide which forms a yellowish complex with molybdate [2]. Briefly, four test tubes were used simultaneously for each sample and designated as blank 1, blank 2, blank 3 and sample tube. Blank 1 tube contained 600 μL of 60 mM sodium potassium phosphate buffer (pH 7.4), blank 2 tube contained 100 μL of 60 mM sodium potassium phosphate buffer and 500 μL of 65 mM urea hydrogen peroxide (pH 7.4), blank 3 contained 100 μL of sample and 500 μL of 65 mM urea hydrogen peroxide, and the sample tube contained the same components as blank 3. Blank 1, 2 and 3 test tubes were incubated at room temperature, while the sample tube was incubated at 37 °C for 1 min. After the incubation step, the reaction was stopped by addition of 500 μL of 32.4 mM ammonium molybdate solution and the absorbance of the yellow complex formed on addition of molybdate was measured at 405 nm using a spectrophotometer against blank 1.

CAT activity was expressed as unit mg^{-1} protein, where 1 unit of CAT was defined as the amount of enzyme that catalysed the decomposition of 1 µmol of hydrogen peroxide per min.

Measurement of glutathione peroxidase (GPx) activity

GPx activity was assessed following a method which is based on oxidation of GSH by H_2O_2 substrate [3], with slight modification. In this assay, glutathione disulfide (GSSG) is immediately converted to GSH in the presence of nicotinamide adenine dinucleotide phosphate (NADPH) which is oxidized to $NADP^+$. A working reagent was first prepared by pipetting 24.8 mL of 50 mM potassium phosphate buffer containing 5 mM EDTA (pH 7.0), 1 mL of 8.4 mM NADPH solution, 1 mL of 0.15 M GSH solution, 100 µL of 113.5 mM sodium azide solution, and 1.1 mL of 38.4 U/mL GR solution into a dark bottle to make a total of 28 mL of working reagent. The solution was vortexed to mix evenly. Next, 260 µL of working reagent was pipetted into separate wells of a clear bottom 96-well plate, followed by the addition of 10 µL of sample (or buffer for blank). The plate was gently shaken for 10 sec before incubation at 37 ^oC for 10 min. Thereafter, 10 µL of 2.2 mM urea H_2O_2 solution was added to initiate the reaction, and the absorbance recorded at 340 nm every min for 4 min using ELISA plate reader. The net absorbance for each sample was obtained by subtracting the absorbance of blank from that of the sample. GPx activity was expressed as unit mg^{-1} protein, where 1 unit of GPx was defined as the amount of the enzyme required to catalyse the oxidation of 1 nmol NADPH per min.

Measurement of glutathione reductase (GR) activity

GR activity was determined using a method which is based on reduction of GSSG in the presence of NADPH which is oxidized to $NADP^+$, with slight modifications [4]. Briefly, 200 µL of 2.728 mM of GSSG solution was pipetted into separate wells of a 96-well plate, followed

by the addition of 20 µL of sample (or buffer, 124 mM potassium dihydrogen phosphate buffer containing 0.62 mM EDTA, pH 7.3 - for blank). The plate was gently vortexed for 10 sec after which it was incubated for 5 min at 37 °C. After incubation, 40 µL of 1.054 mM of NADPH solution was added to initiate the reaction. The decrease in absorbance was measured at 340 nm every min for 4 min using ELISA plate reader. The net absorbance for each sample was obtained by subtracting the absorbance of blank from that of the sample. GR activity was expressed as unit mg^{-1} protein, where 1 unit of enzyme was defined as the amount of enzyme required for the oxidation of 1 nmol of NADPH per min.

Measurement of glutathione (GSH) level

GSH level was measured following the method of Jollow, et al. [5], which is based on the reduction of 5-thio-2-nitrobenzoic acid formed by the reaction of 5,5-dithiobis-2-nitrobenzoic acid (DTNB) with the sulfhydryl group of GSH. Samples were first deproteinised to eliminate protein interference with the assay. To deproteinise the samples, 100 µL of each sample was pipetted into a 1.5 mL Eppendorf tube, followed by the addition of 100 µL of 10 % (w/v) metaphosphoric acid. The content was vortexed for 10 sec and allowed to stand at room temperature for 5 min, followed by centrifugation at 1200 g for 15 min at 4 °C. 100 µL of the supernatant was pipetted into a separate 1.5 mL Eppendorf tube, followed by the addition of 5 µL of 4 M triethanolamine solution, and vortexed to mix. The samples were kept at -80 °C until use. This procedure was also performed for the diluted standards to eliminate the need for any dilution factor.

To assay for GSH level in deproteinised samples, 10 µL of samples and standards were pipetted into separate wells of a 96-well plate, followed by the addition of 140 µL of working reagent. The plate was vortexed for 5 sec, followed by the addition of 10 µL of 1 mM DTNB solution. The increase in absorbance was measured at 412 nm every min, for 5 min, using

ELISA plate reader. The net absorbance for each sample was obtained by subtracting the absorbance of blank (0.2 M potassium phosphate buffer containing 0.01 M EDTA, pH 7.5) from that of the sample. Total GSH level in each sample was estimated from a 6-points standard curve (0 – 25 µg/mL) obtained using GSH standard solutions, and results were normalised with protein concentration, and expressed as nmol GSH equivalent mg^{-1} protein.

Measurement of malondialdehyde (MDA) level

MDA level was measured as a marker of lipid peroxidation using the method of Ohkawa, et al. [6]. Briefly, 1.5 mL of glacial acetic acid (pH 3.5), 200 µL of 8.1 % (w/v) sodium dedocyl sulphate, 1.5 mL (w/v) of 2-thiobarbituric acid (TBA) solution, 700 µL of distilled water and 100 µL of sample, standard (1,1,3,3-tetraethoxypropane solution, TEP) or blank (0.1 M Tris-HCl solution, pH 7.4) were pipetted into a test tube, vortexed and kept in boiling water bath (95 ^{o}C) for 1 h with a marble on top of each test tube. After cooling in ice bath, the test tubes were centrifuged at 3000 g for 10 min at 25 ^{o}C. Following centrifugation, 1 mL of the supernatant was pipetted into a cuvette and the absorbance was read at 532 nm using a spectrophotometer. The net absorbance for each sample was obtained by subtracting the absorbance of blank (0.1 M Tris-HCl solution, pH 7.4) from that of the sample. The level of MDA was determined from a standard curve derived using TEP standard solutions (0, 2, 4, 6, 8 and 10 µM), and expressed as nmol MDA Eq mg^{-1} protein.

Measurement of total antioxidant capacity (TAC)

TAC was determined following a method which is based on suppression of thiobarbituric acid reactive substances (TBARS) production after the reaction involving Fe-EDTA complex and hydrogen peroxide [7]. The inhibition of colour development (suppression of TBARS production) was measured spectrophotometrically at 532 nm. In this assay, each

sample (designated A_I) had its own control (designated A_O) in which the sequence of the addition of reagents to its tube was different from that of the actual sample tube. Also, a negative control (N_I and N_O) and uric acid standard (UA_I and UA_O) were included and the sequence of reagent addition was same as in A_I and A_O, except that the sample was replaced with 100 mM sodium phosphate buffer (pH 7.4) for negative control and uric acid (1000 µM) for standard.

Briefly, 500 µL of 100 mM sodium phosphate buffer (pH 7.4), 500 µL of 10 mM sodium benzoate, 200 µL of Fe-EDTA complex solution, 15 µL of sample (100 mM sodium phosphate buffer, pH 7.4 in the case of negative control, or 1000 µM uric acid in the case of standard) and 200 µL of 10 mM urea hydrogen peroxide solution were added to a test tube designated A_I (for sample tube), N_I (for negative control tube) and UA_I (for uric acid standard tube). Thereafter, the test tubes were vortexed and incubated for 1 h at 37 °C (first incubation). After the first incubation, 1 mL of 20 % (v/v) acetic acid was added to the test tubes, followed by incubation in a water bath at 100 °C for 10 min (second incubation). The test tubes were then cooled in ice bath. One mL of the mixture in each test tube was transferred into a cuvette and the absorbance was read at 532 nm using a spectrophotometer against distilled water blank. For tubes A_O, N_O and UA_O, similar protocol as described above was employed, except that 20 % (v/v) acetic acid was added before the first incubation. TAC for each sample was calculated using the formula below and expressed as µmol of UA equivalent mg^{-1} proetin.

TAC (µmol UA equivalent mg^{-1} protein) =

$$\frac{N - A}{N - UA} \times \frac{\text{UA concentration}}{\text{Protein conc. (mg L}^{-1}\text{)}}$$

Where,

N = absorbance of negative control ($N_I - N_O$)

A = absorbance of sample ($A_1 - A_O$)

UA = absorbance of uric acid standard solution ($UA_1 - UA_O$)

UA concentration = 1000 μM

References

1 Al Batran, R. *et al.* In vivo antioxidant and antiulcer activity of *Parkia speciosa* ethanolic leaf extract against ethanol-induced gastric ulcer in rats. *PloS One* **8**, e64751 (2013).

2 Goth, L. A simple method for determination of serum catalase activity and revision of reference range. *Clinica Chimica Acta* **196**, 143-151 (1991).

3 Paglia, D. E. & Valentine, W. N. Studies on the quantitative and qualitative characterization of erythrocyte glutathione peroxidase. *Joural of Laboratory and Clinical Medicine* **70**, 158-169 (1967).

4 Carlberg, I. & Mannervik, B. in *Methods in Enzymology* Vol. 113 484-490 (Elsevier, 1985).

5 Jollow, D. J., Mitchell, J. R., Zampaglione, N. & Gillette, J. R. Bromobenzene-induced liver necrosis. Protective role of glutathione and evidence for 3,4-bromobenzene oxide as the hepatotoxic metabolite. *Pharmacology* **11**, 151-169 (1974).

6 Ohkawa, H., Ohishi, N. & Yagi, K. Assay for lipid peroxides in animal tissues by thiobarbituric acid reaction. *Analytical Biochemistry* **95**, 351-358 (1979).

7 Koracevic, D., Koracevic, G., Djordjevic, V., Andrejevic, S. & Cosic, V. Method for the measurement of antioxidant activity in human fluids. *Journal of Clinical Pathology* **54**, 356-361 (2001).
